# Building the foundation for comprehensive suicide prevention – based on intention and planning in a social–ecological context

**DOI:** 10.1017/S2045796019000659

**Published:** 2019-11-08

**Authors:** Eric D. Caine

**Affiliations:** Center for the Study and Prevention of Suicide, University of Rochester Medical Center, Rochester, NY 14642, USA

**Keywords:** Health outcomes, mental health, social and political issues, suicide

## Abstract

National suicide prevention programmes that have been successful in reducing rates or keeping them low have been intentional, with collective alignment of local, regional and national priorities. Prevention efforts must begin well before individuals become suicidal, complementing readily available clinical services that address the needs of acutely distressed persons. These efforts, which focus on the antecedent risks and vulnerabilities of key populations, have the potential to diminish premature mortality from multiple causes, even as reducing suicide is the outcome of primary interest. In this commentary, I consider four key challenges that must be confronted in order to develop effective, broadly reaching systemic strategies that, at once, can be adapted locally while being implemented nationally – challenges that are framed in a social–ecological context. They involve defining the scope of the problem, meeting essential data needs, developing and modelling measurable implementation strategies and building prevention efforts based on shared culture and values.

Suicide is preventable – as shown through dramatic changes in countries such as Denmark and Finland, and relatively low, sustained rates in England (OECD, 2019). Each country instituted tailored strategies that combined multiple elements – reducing or constraining access to lethal methods, such as removing carbon monoxide from domestic gas, taking co-proximal off the market and limiting the package size of paracetamol; enhancing access to mental health and crisis services; and building a collective approach to awareness and safety. Some of these services were government sponsored; e.g. Denmark's Suicide Prevention Clinics. Others have been implemented by non-governmental organisations, such as the Samaritans (England), Lifeline (Denmark) and Mental Health Finland.

While these intentional efforts were being implemented in economically resourced countries, suicide rates in many countries declined – largely based on the benefits of economic development in many nations and the reduction of poverty (WHO, 2014). However, countries such as the United States (US) and Australia (AU) now are seeing rising rates – the former having steady increases since 2000 (OECD, 2019). Unlike Denmark and Finland, which have populations the size of US states, or England, which has a long tradition of public health interventions, the US and AU have huge land masses; multi-ethnic and multi-racial populations with distinctive cultures and federal systems that depend on the concerted actions of states, and regional and local authorities for the implementation of nationally important initiatives. AU now is in the midst of a series of trials, sponsored by a combination of national, provincial and private grants. Whether these succeed in the near-term, they are building the *collective alignment* necessary for longer-term success. Such alignment of efforts has been essential to the success of effective, intentional, comprehensive prevention programmes; however, achieving this necessary clarity of vision and unity can be especially challenging in countries (and cultures) that value local control and individual self-sufficiency.

In this commentary, I consider four key challenges that must be confronted in order to develop effective, broadly reaching strategies that, at once, can be adapted locally while being implemented nationally. These are framed in a social–ecological context that integrates individuals into family, community and larger societal perspectives (see Fig. 1) (Caine, 2013; Health Foundation, 2014; Furst *et al*., 2019).

**Fig. 1.**
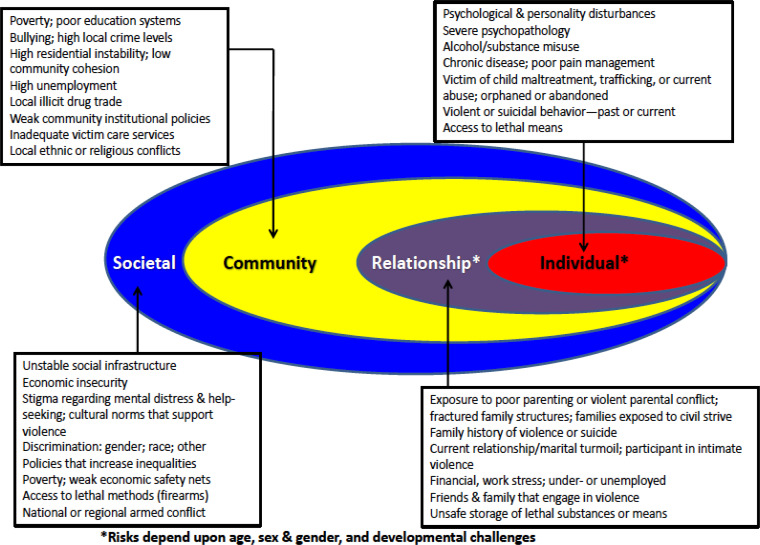
Ecological Model: Mental health & social risks for violence to self and others (modified from Caine, 2013).

While it may be possible to deploy initiatives that have potent local effects, suicide prevention efforts intended to change death rates in nations must *intentionally* address or mitigate the impact of adverse or exacerbating external forces, such as economic downturns, and foster a commonly adopted local, regional, national commitment to actions that promote healthy development and safe environments. Prevention begins long before someone becomes suicidal and involves policies and actions that address both community and individual needs. As I consider these foundational challenges, I will not recapitulate other recent publications and reports that discuss many step-by-step actions forming collaborative community efforts (Stone *et al*., 2017; Caine *et al*., 2018; WHO, 2018). Rather, my intention is to complement current perspectives by raising fundamental issues not often considered elsewhere.

*Challenge 1. Defining the scope of prevention*: It is important to distinguish between suicide prevention and the clinical care of suicidal persons, most of whom ultimately do not die by suicide (Bergen *et al.*, 2012). ‘Suicide prevention’ in the US focuses on detecting and treating suicidal persons (Sall *et al*., 2019); no doubt, these individuals are in great distress and need care. However, prevention requires recognising and mitigating antecedent risks among populations and groups whose adverse life experiences and day-to-day behaviours set the stage for declining health and mental health, leading to premature mortality from self-injury fatalities including suicide and drug-ingestions and from ‘natural causes’ – e.g. vascular, hepatic, gastrointestinal and infectious diseases, and cancers. If prevention, in fact, precedes the time when a person becomes suicidal or makes a fatal attempt, outcomes that we measure should involve the reduction of premature deaths from multiple risk-related causes, even as suicide is our primary interest. Notably, Denmark, Finland and England have health systems and prevention efforts that broadly address population health priorities. This broader scope of action fosters universal preventive interventions that are essential to suicide prevention, even as they were not conceived for this purpose.

*Challenge 2. Data, data, data*: Key data are essential for defining the burdens of suicide, developing a strategic vision, designing specifically targeted programmes and monitoring near-term effects (e.g. referrals to care; attempts; unanticipated consequences) and distal outcomes (e.g. premature deaths due to risk-selected natural causes, suicides, drug-intoxication) (McGrath *et al*., 2018). One example for geospatial burden mapping is found at the Colorado Department of Public Health and Environment (https://cohealthviz.dphe.state.co.us/t/HSEBPublic/views/CoVDRS_12_1_17/Story1?:embed=y&:showAppBanner=false&:showShareOptions=true&:display_count=no&:showVizHome=no#4), a data dashboard using state information reported to the US National Violent Death Reporting System. Now the US Centers for Disease Control and Prevention (CDC) is building capacity as part of the National Syndromic Surveillance Program, developed originally for detecting emerging infectious diseases, to report local emergency department admissions weekly for suicide attempts and opioid drug ingestions. Moreover, as suicide is a lower frequency event, it is incumbent on planners to identify ‘intermediate’ outcomes that are on the path to possible suicide – outcomes that are more numerous and accessible to meaningful measurement.

Such data provide the basis to forge local action-coalitions. Seeing before you the cumulative impacts of suicide and risk-related outcomes in your community reduces the abstraction when looking at national or state-level reports. Locally culled data also confront planning participants with the heterogeneity of suicide and attempted suicide. Many policy-drivers understandably carry personal ‘sample biases’, based on work experiences, family losses or inherent priorities. For example, it is common in the US to hear an over-riding emphasis on suicide prevention among youth, driven by the commitment of parents who have lost children. The availability of robust, reliable data serves to open the door for consideration of all who are affected.

*Challenge 3. Developing and modelling measurable implementation strategies*: Individually focused interventions (i.e. one-by-one) that characterise most prevention efforts in the US have yet to affect the rising rate of suicide. While there are occasional, encouraging publications of apparently successful public health approaches, there have yet to be scalable, comprehensive initiatives – let alone, measured outcomes. Recently, authors have applied system dynamic modelling methods to simulate the impacts of multi-component programmatic efforts (Atkinson *et al*., 2019*a*, 2019*b*). While promising, this methodology will require a much more diverse set of input sources and measures, including metrics regarding societal and community factors and values, available (potentially overlapping) social support and legal measures that might be implemented, and a deeper understanding regarding the placement and integration of primary care, acute care and follow-up mechanisms. The availability of community-based mental health services has been key for successes in Denmark and Finland (Pirkola *et al*., 2009; Erlangsen *et al*., 2015); these must be geospatially mapped and developed to assure the well-placed availability of early-intervention, acute service and follow-up care that is requisite for assuring sustained attention to those identified as pre- or peri-suicidal (Chung *et al*., 2018). Service mapping and mapping of burdens are tightly linked. While broadly based approaches are central to reducing the number of persons ‘coming to suicidal’, selective and indicated preventive services are required to care for vulnerable persons and those with acute needs, and to boost efforts to reduce mortality (Caine, 2013).

*Challenge 4: Culture and values*: Collective alignment of values and actions has been central to the success of intentional prevention programmes that are both far-reaching (systemic) and implemented in an organised, sustained fashion (systematic). Understanding suicide as a *public* health problem – indeed, a public health crisis in many countries – implies larger public responsibility that reaches beyond individuals and families. Such crises require definitive actions from governmental agencies, as well as voluntary, non-government organisations and they are premised on the (potentially) intrusive notion that these deaths are not solely a private matter.

For countries that have more collective cultures, many of these issues have been resolved in favour of expecting that their society, their government and allied community agencies will create together a web of complementary prevention programmes. The high expectations for such services are balanced by a general acceptance that, to some extent, social organisations reach into the lives of families and individuals in order to provide timely and effective support.

In a country such as the US, there is constant flux and tension between values that promote individual responsibility and those that advocate collective concern and care. It remains to be seen whether the crisis of rising suicide rates in the US will prompt the kind of discussion necessary to fully commit to collective actions. This type of commitment serves as the predicate for building the foundation for effective public health programmes that require more than offering a single-dose vaccination – or removing a pump handle (even as the London cholera epidemic may have been waning). Once there is such commitment – the permissive green light for action – defining the scope of the problems, digesting and using data, and forging far-sighted, ambitious strategies becomes possible.
